# Antimicrobial Polymer-Based Hydrogels for the Intravaginal Therapies—Engineering Considerations

**DOI:** 10.3390/pharmaceutics13091393

**Published:** 2021-09-02

**Authors:** Monika Gosecka, Mateusz Gosecki

**Affiliations:** Polymer Division, Centre of Molecular and Macromolecular Studies, Polish Academy of Sciences, Sienkiewicza 112, 90-363 Lodz, Poland; gosecki@cbmm.lodz.pl

**Keywords:** antibacterial, antifungal, anti-*Trichomonas vaginalis*, drug delivery system, gynaecological therapy, intravaginal treatment, unimolecular micelles, nanoparticles, microparticles, bigel, injectable hydrogel, thermogelling polymers

## Abstract

The review is focused on the hydrogel systems dedicated to the intravaginal delivery of antibacterial, antifungal and anti-*Trichomonas vaginalis* activity drugs for the treatment of gynaecological infections. The strategies for the enhancement of the hydrophobic drug solubility in the hydrogel matrix based on the formation of bigel systems and the introduction of nano- and microparticles as a drug reservoir are presented. Hydrogel carriers of natural and synthetic pharmacological substances, drug-free systems displaying antimicrobial activity thanks to the hydrogel building elements and systems combining the antimicrobial activity of both drug and polymer building components are distinguished. The design of hydrogels facilitating their administration and proper distribution in the vaginal mucosa and the vagina based on thermoresponsive systems capable of gelling at vaginal conditions and already-cross-linked injectable systems after reaching the yield stress are discussed. In addition, the mechanisms of hydrogel bioadhesion that regulate the retention time in the vagina are indicated. Finally, the prospects for the further development of hydrogel-based drug carriers in gynaecological therapies are highlighted.

## 1. Introduction

Vulvovaginitis is one of the most frequent gynecological diseases [1,2]. Vulvovaginal infections are most commonly caused by vulvovaginal candidiasis, trichomonal vaginitis, and bacterial vaginosis [3]. In 2019, from 5 to 70% of women worldwide suffered from bacterial vaginosis [4]. Vaginal candidiasis most often belongs to the *Candida albicans* infections [5]. Other strains, such as *Candida tropicalis*, *Candida* *parapsilosis*, *Candida* *crusei*, *Candida glabrata*, *Candida* *stellatoidea*, and *Candida* *lusitaniae,* cause infections as well. Almost 75% of women suffer from candidiasis at least once in their life [5]. *Trichomonas vaginalis* is a human protozoan pathogen responsible for the most common non-viral sexually transmitted disease worldwide [6]. Moreover, infections with multiple pathogens simultaneously have been observed.

Therapies based on oral drug administration are usually effective; however, there are numerous systemic adverse effects associated with their usage. For example, oral administration of metronidazole, besides causing nausea, headache, insomnia, dizziness, and dry mouth, leads to leukopenia and neutropenia [7]. Auranofin, an anti-*Trichomonas* agent, is toxic due to its long plasma half-life (35 days) [8], resulting in sustained systemic exposure in spite of the short period of oral administration [6]. Another example is an antifungal drug, miconazole nitrate, that causes a blood disorder, thrombocytopenic purpura [9]. In general, oral drug administration is problematic for women suffering from gastrointestinal tract disorders as well as pregnant women, for whom such therapies are especially dangerous taking into account the fetus and its proper development.

To prevent systemic drug toxicity, gynaecological treatment routinely employs topical therapies to deliver the drug locally to the affected area in vaginal suppositories. Unfortunately, such therapy is very often not effective. Due to frequent uncontrolled leakage of the suppository, the retention time of the content in the target tissue is insufficient. In fact, the amount of absorbed drug, as well as the part of the tissue exposed to the drug, is often unknown. This necessitates frequent drug administration, up to several times a day, which is highly uncomfortable for patients, especially those who are professionally active. In addition, the total dose of administered drug must be high. As a result, the lack of control over drug delivery may lead to the reoccurrence of infections [10] or even to the development of drug resistance. Ineffective treatment leads not only to chronic inflammation but also to miscarriage or even infertility. Conventional vaginal suppositories do not fulfil their function as a result of poor adhesiveness and a short retention time [11]. Due to the short period of residence in the genitourinary tract, multiple and frequent drug administration is required. To address these challenges, hydrogel formulations have been designed to improve drug delivery [12,13]. This results from their compatibility with aqueous environments, biocompatibility, high porosity, and, in addition, their capacity for controlled drug delivery [14]. Hydrogels are a suitable matrix for the controlled delivery of biologically active substances thanks to their mechanical properties and controlled drug release capability [15]. Moreover, the prolonged contact of the formulation with the vaginal mucosa, in comparison to a solid suppository, increases the drug efficacy.

The most suitable hydrogel system for gynaecological treatment, however, should exhibit the property of shape-conforming. This would facilitate the continuous coverage of the afflicted area and would prolong the retention time of the pharmacological formulation in the vaginal mucosa. Such a gel is not easily removed by the self-cleansing mechanism. The uniform distribution of the drug-carrying hydrogel and the sustained release of the drug provide the required drug concentration in the vaginal mucosa and therefore are of great importance for the performance of vaginal therapies. Traditional hydrogels, i.e., those based on irreversible covalent cross-links, fail as the cross-linking process defines their shape at the synthesis step and a proper fit to the tissue surface is impossible. In addition, the complete removal of the hydrogel carrier is problematic. The most appropriate hydrogel systems are those constructed on reversible cross-links, i.e., reversible covalent linkages [16,17,18] and supramolecular interactions [19,20,21,22], which display responsive behaviour toward external stimuli. Dynamic hydrogel systems constructed on adaptable linkages, i.e., reversible covalent or supramolecular bonds, which are broken and reformed in a reversible manner without the usage of external triggers, exhibit desirable rheological properties that ensure hydrogels’ injectability and their ability to form a continuous film on the infected site.

Another important issue regarding hydrogel systems as gynaecological drug carriers is the discrepancy in the hydrophilicity of hydrogels and drugs commonly used in gynaecological treatment. These drugs are simply poorly soluble in a hydrogel matrix. Nonetheless, the controlled administration of water-insoluble drugs is of great importance as it reduces the amount of drug administered and, in consequence, it limits drug-related toxicity and side effects.

In this paper, we present the strategies for the construction of hydrogels suitable for gynaecological drug delivery systems with the usage of different polymer systems. This review is divided into four sections dealing with the aspects of the intravaginal administration of the hydrogel platform, the methods of hydrophobic drug solubility in the hydrogel matrix, drug-free pharmacological hydrogel materials displaying antimicrobial properties, and bioadhesion mechanisms between hydrogel and vaginal mucosa.

## 2. The Design Strategies of Hydrogel Systems to Facilitate the Efficient Coverage of the Afflicted Vaginal Area

The key factor in the design of an ideal intravaginal hydrogel is its consistency, which should facilitate convenient administration to the target site, ensuring even distribution of the formulation on the mucosal tissue. Such a formulation should ensure adequate coverage of the vagina thanks to high spreadability, which, along with the mucoadhesion of the formulation, determines the therapy’s efficacy.

**Scheme 1:**
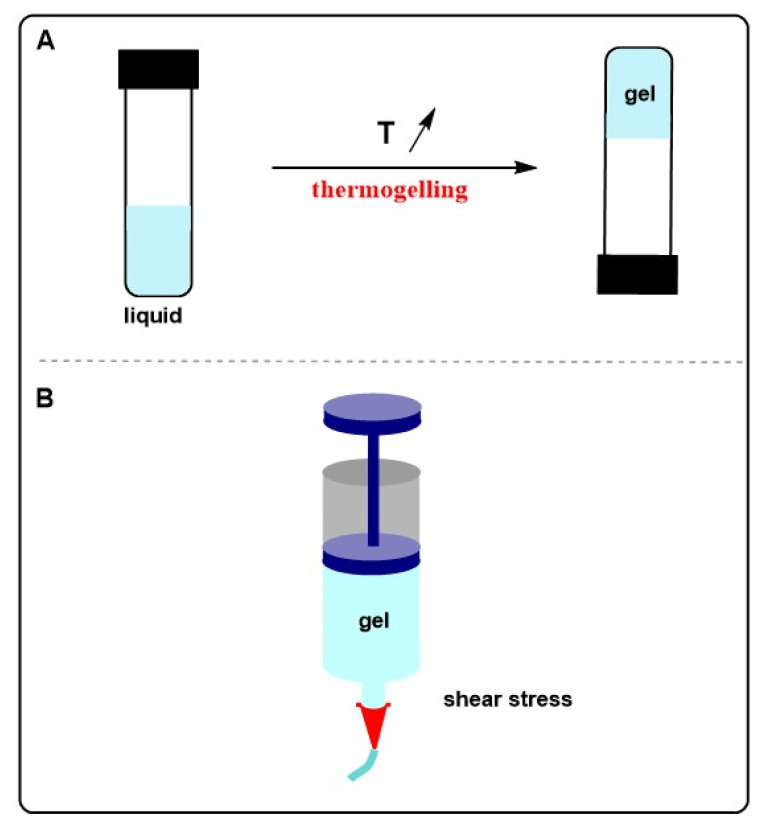
Mechanisms of intravaginal hydrogel administration: thermogelling behaviour of polymer solution upon heating at vaginal conditions (**A**), and hydrogel injection at the shear stress above the shear yield (**B**).

Intravaginal hydrogel-based drug carriers can be formed in situ upon incorporation into the vagina or a pre-formed hydrogel system can be injected to the target site. The first group of hydrogels relates to the thermogelling-based aqueous solutions of polymers. Such systems are aqueous solutions (liquid-like behaviour) at room temperature and, after administration into the vagina, upon the increase in temperature, undergo a transition from liquid to a semi-solid or solid gel (Scheme 1A). In the case of very low-viscosity solutions, instead of liquid injection, the formulation can be applied in the form of an aerosol [23]. In addition, the introduction of a propellant (propane/butane, 80:20 *v*/*v*) into the polymer solution results in the formation of an expansible thermal gelling foam aerosol [24].

The second class of hydrogels are systems whose molecular structure facilitates injectability above the yield stress, i.e., shear stress at which the viscosity of the material significantly decreases, as an effect of the network degradation (Scheme 1B). After administration, the network is reorganised thanks to the dynamic nature of the cross-links and the hydrogel recovers the initial rheological properties, i.e., before injection [25].

### 2.1. Thermogelling Polymer Systems

**Scheme 2:**
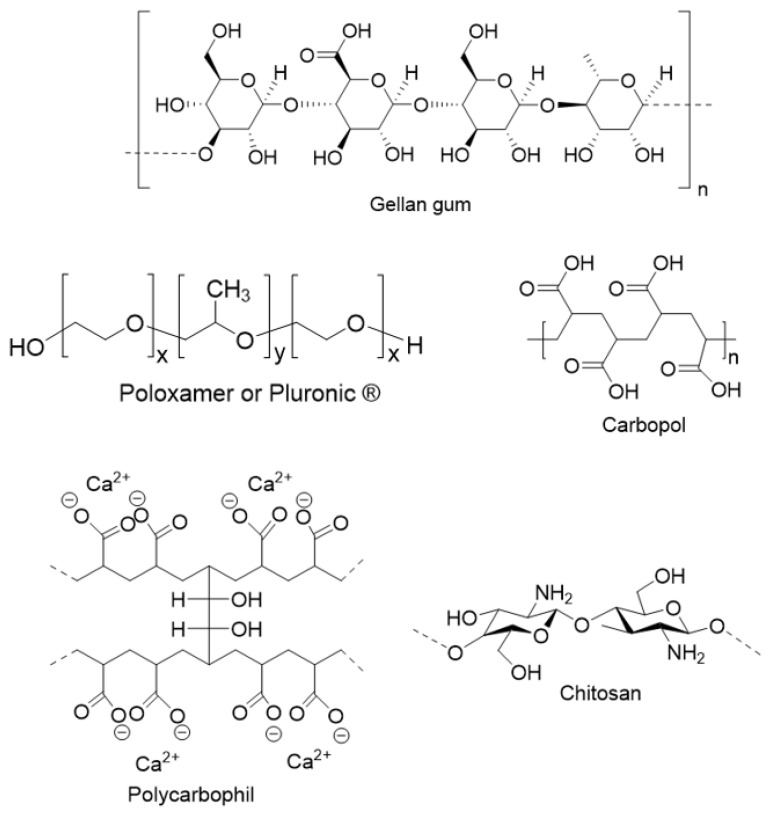

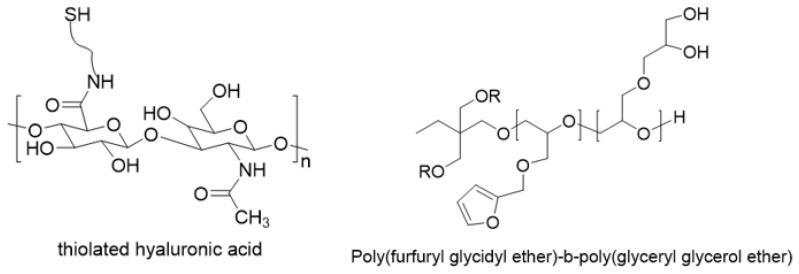
The molecular structure of most polymers applied for the formation of hydrogels dedicated to gyneacological therapies.

Both synthetic and natural polymers of thermoresponsive properties are used to form hydrogels dedicated to gynaecological treatment. Amongst synthetics formulations, Poloxamer^®^ 407, composed of a poly (propylene oxide) central block and hydrophilic poly (ethylene oxide) blocks (Scheme 2), can be distinguished; an 18 wt% aqueous solution at 4–5 °C turns into a hydrogel at 32 °C [23,26,27]. Due to the copolymer’s amphiphilic nature, aqueous solutions at critical micellar concentration and temperature form micelles as an effect of dehydration of the hydrophobic poly (propylene oxide) block. For a sufficiently concentrated polymer sample, the arrangement of micelles results in its gelation. Analogous poly (ethylene oxide)-b-poly (propylene oxide)-b-poly (ethylene oxide) (PEO-PPO-PEO) block copolymers, Pluronic^®^ F-127 (PF-127) and Pluronic^®^ F-68 (PF-68), which differ in the molecular weight and molar ratio of PEO to PPO, exhibit thermoresponsiveness [28]. The average molecular weight of PF-127 was 12,600 g/mol and the PEO/PPO ratio was 7:3, whereas for PF-68, the PEO/PPO ratio was 8:2 and the molecular weight was 8400 g/mol. The adjustment of the rheological properties of formed hydrogels suitable for gynaecological administration actually required the usage of both PF-68 and PF-128 copolymers, as separate usage of the components led to the formation of a weak gel or an overly hard hydrogel [28]. Hydrogels for gynaecological therapies based on amphiphilic copolymers of poly (propylene oxide) blocks and poly (ethylene oxide) blocks were applied for the controlled delivery of synthetic antimicrobial drugs such as metronidazole [29], clotrimazole [28], amphotericin [23], and amoxicillin [26]. Clotrimazole and amphotericin displayed antifungal activity, whereas amoxicillin is an antibacterial drug. Metronidazole has a wider spectrum against microbes, as it displays both antibacterial and anti- *Trichomonas vaginalis* action.

Another polymer system prone to rapid temperature-induced gelation and dedicated to gynaecological antimicrobial treatment is nature-derived chitosan [30] (Scheme 2) in combination with β-glycerolphosphate [6]. The mechanism of thermoresponsive gelation is a result of the strengthening of hydrophobic interactions upon an increase in temperature. Heat induces the transfer of protons from chitosan to glycerol phosphate, thereby neutralising chitosan and allowing attractive interchain forces to form a physical gel [31]. At low temperatures, the interactions between chains are impaired as a result of strong polymer interactions with water. Zhang et al. prepared auranofin-loaded β-glycerolphosphate and chitosan-based hydrogel at a low polymer concentration for the treatment of *Trichomonas vaginalis* [6]. Upon heating from 20 to 37 °C, both G’ and G” increased within 1 min, and these moduli did not change within the frequency range of 10 to 1 Hz. A hydrogel based on β-glycerolphosphate and chitosan was also found to be suitable to deliver amoxicillin, as an antibacterial drug carrier for gynaecological treatment [26].

### 2.2. Injectable Hydrogel Systems

Besides thermogelling mixtures with β-glycerolphosphate, chitosan is able to form a hydrogel on its own at acidic pH [30]. Chitosan-based hydrogels for vaginal applications are prepared by dissolving it in 5% *v*/*v* acetic with further pH adjustment to 5.0 [32]. The viscosity of such hydrogels drops at high shear stress and the material behaves like a fluid. The yield stress is a material parameter that determines the shear stress, at which the material loses its solid behaviour and starts to flow. This means that the prior-formed hydrogel platform that exhibits yield stress may be injectable. The application of injectable gel requires, however, the usage of higher stress than in the case of a solution, in order to be implemented at the target site. Chitosan-based hydrogels were applied for vulvovaginal candidiasis treatment [32,33] bearing *Mitracarpus frigidus* extract [32] and *Pelargonium graveolens* essential oil [33].

Gellan gum, a natural anionic polysaccharide obtained via fermentation of the *Sphingonomonas elodea* microorganism, in which the repeating unit is a tetrasaccharide composed of α-l-rhamnose, β-d-glucose, and β-d-glucoronate in the molar ratio 1:2:1 [34] (Scheme 2), thanks to the presence of anionic carboxyl moieties in β-d-glucoronate units, forms electrostatic interactions with cations. Gellan gum was applied for the formation of a hydrogel system by cross-linking with cationic polymer Eudragit^®^ RS 100-enriched nanocapsules for the delivery of indole-3-carbinol, a drug for the treatment of trichomoniasis [35]. Under conditions of high shear rates, the hydrogel was able to flow, which facilitated its target application and spreadability.

Carbopol, a high-molecular-weight poly (acrylic acid) cross-linked with allyl ethers of pentaerythritol (Scheme 2), is a synthetic polymer that has found application in gynaecological hydrogel formulations [36]. At acidic conditions, it displays low viscosity; however, upon neutralisation, this polymer forms a highly viscous gel [37], displaying injectable behaviour. A Carbopol-based hydrogel bearing metronidazole was applied in the treatment of bacterial vaginosis [36].

Polymers enriched with thiol groups found application in hydrogel formation for gynaecological therapies via their cross-linking with disulphide linkages. This approach was used for a mixture of 8-arm poly (ethylene glycol) bearing thiol terminating groups and 4 poly (amidoamine) dendrimer equipped with peripheral thiopyridyl end groups, which resulted in the formation of injectable hydrogel thanks to intramolecular disulphide cross-links [38]. The hydrogel construction facilitated the entrapment of amoxicillin, and thus the formed platform displayed antibacterial properties. The residence time of the hydrogel was 72 h. The hydrogel was well-tolerated by tissue. This therapeutic platform is suitable for intravaginal treatment in pregnant women as the dendronised hydrogel building component does not cross the human fetal membranes. Another usage of thiol moieties relates to the Michael reaction by cross-linking with the vinyl end groups of PEG [39,40] or in the reaction of thiolated carboxymethyl hyaluronic acid with poly (ethylene glycol)-bisbromoacetate [41]. The latter system was applied to obtain dry film enriched with metronidazole, which, after administration in the vagina, was rehydrated.

Vicinal shell diol moieties of the unimolecular micelles based on star-shaped poly (furfuryl glycidyl ether)-b-poly (glycidyl glycerol ether), (Scheme 2) were applied to form reversible boronic ester cross-links with 2-acrylamidephenylboronic acid moieties incorporated into the acrylamide copolymer [42]. On one hand, the boronic esters are sensitive to an increase in temperature, as the process of their formation is exothermic. This means that heating the boronic ester-based hydrogel leads to the loosening of its structure, i.e., to a more liquid-like behaviour. On the other hand, upon the usage of higher shear stress, i.e., attaining the yield stress value, the hydrogel is injectable, as the viscosity of the material immediately decreases and it displays liquid-like properties.

## 3. The Strategies for the Improvement of Hydrophobic Drugs’ Solubility within Hydrogel Systems for Intravaginal Therapies

An ideal delivery system for gynaecological treatment should ensure adequate drug concentrations at the site of infection for a sufficient period of time. In order to achieve high therapeutic efficacy in the treatment of microorganism-originating inflammation, satisfactory drug solubilisation is crucial. Upon hydrophobic drug solubilisation, its bioavailability become high enough to provide in vivo effectiveness. Insufficient drug solubility in the hydrogel platform, besides reduced bioavailability, can lead to the discontinuity of the gel structure as an effect of the presence of precipitated drug particles. As a result, the uncontrolled fracture of the hydrogel platform can be observed. This problem can be even serious upon the dehydration of the material. The prepared films are difficult to be used for intravaginal administration due to their fragility [41].

Due to the hydrophilic nature of hydrogels, it is essential to implement proper strategies to enhance drug solubility in the hydrogel matrix. The most common consists in the application of block amphiphilic copolymers [43], which, at concentrations above the critical micellar concentration, exist in the form of micelles. On one hand, hydrophobic segments in the inner part of the micelle improve the solubility of poorly soluble drugs in water. On the other hand, hydrophilic moieties ensure solubility in water to the drug-loaded macromolecular constructs. The gold standard for block amphiphilic copolymers are poly (ethylene oxide)-b-poly (propylene oxide)-b-poly (ethylene oxide) (PEO-PPO-PEO) block copolymers. The limitation of such micelles, however, is that the dilution of this system leads to their disassembly. More controlled systems are unimolecular polymer micelles, composed of a hydrophobic core and hydrophilic shell, which are covalently bound. Recently, our group demonstrated the efficient solubility of nifuratel, a drug with a broad antimicrobial spectrum of action, by the usage of star-shaped poly (furfuryl glycidyl ether)-block-poly (glyceryl glycerol ether) macromolecules [42]. The suitably chemically adjusted core to a drug, according to the principle “like dissolves like”, ensures a high level of drug solubilisation. In this case, the furan-rich core enhanced the solubility of nifuratel, in whose structure a nitrofuran moiety is present. The hydrophilic macromolecule corona ensure the water solubility of the whole construct. In addition, the diol-rich shell of the construct introduces the possibility of its dynamic cross-linking with poly (boronic acids), which ensures the injectability and self-healing of the hydrogel.

Another strategy for drug solubility improvement consists in the formation of bigel systems, i.e., the combination of organogel domains in the hydrogel matrix. In this way, metronidazole was dissolved in a sorbitan monostearate–sesame oil organogel, which, in the molten state, was dropped into a Carbopol^®^ 934 hydrogel at 60 °C [36]. The size of droplets increased proportionally with the fraction of the organogel and was in the range of 10 to 35 µm. The obtained bigel systems exhibited shear-thinning, were viscoelastic in nature, and displayed satisfactory activity against *Escherichia coli*. It is noteworthy, however, that the control of drug release was strictly dependent on the ratio of organogel to the hydrogel fraction. The fraction of organogel must be high enough to attain controlled drug release. This results from the fact that organogel is a drug reservoir. In the case of the bigel composed mainly of hydrogel (90 wt%), almost the entire amount of the entrapped drug was released in 1.5 h. The increase in the organogel fraction in the bigel system to 19 wt% resulted in the release of 80% of metronidazole in 12 h.

The combination of the hydrogel system with another drug carrier ensures sustained drug release. It can be attained by the incorporation of nano- or micro-objects, which, thanks to the improvement of drug solubility, play the role of a reservoir for drug molecules. The direct usage of particles for efficient vaginal treatment is impossible, due to their insufficient retention time in the mucosa [6]. Their introduction into the structure of the hydrogel system not only ensures the incorporation of a substantial amount of hydrophobic drug into the hydrogel material and prolonged contact time at the site of drug action, but the release rate can be also adjusted with the properties of both carriers. Amongst particles applied for the enhancement of vaginal treatment, nanosponges [28,44], liposomes [45,46], nanocapsules [35], and solid particles [6], etc., can be distinguished.

Nanosponges are an interesting class of particles equipped with numerous nanometre-wide cavities that are able to enhance hydrophobic drugs’ solubility and prolong the drug release. By altering the pharmacokinetic parameters, they improve drug bioavailability [44,47] and thus the efficacy of the therapy. Osmani, et al. used highly swollen nanosponges composed of hyper cross-linked hydroxypropyl β-cyclodextrin-based (HP-β-CD) colloidal structures with three-dimensional networks in order to increase clotrimazole’s solubility [28]. The enhanced drug solubility resulted from the cyclodextrin’s ability to form inclusion complexes with hydrophobic drugs in water. The cyclodextrin-based nanosponges exhibited high drug loading and controlled drug release. Their embedment in a Pluronics^®^ F-127/F-68-based hydrogel resulted in almost complete drug release within 15 days of the beginning of the experiment, along with only a few signs of inflammation after the treatment with a nanosponge–hydrogel complex platform in a histopathological study. For neat hydrogel, i.e., without nanosponges, saturated with clotrimazole, cumulative drug release was observed in 6 h. Both neat and nanosponge-enriched hydrogels containing clotrimazole were well tolerated by rats, without signs of erythema and oedema. An in vivo irritation study did not show any irritant effect.

Widely applied solid nano- and microparticles have also found usage for the formation of a hybrid drug carrier. Zhang et al. prepared 130 nm poly (lactic-co-glycolic) acid (PLGA) particles loaded with auranofin. The particles were embedded in a thermoresponsive mixture of β-glycerolphosphate and chitosan, which resulted in even drug distribution on the vaginal mucosa but also in sustained drug release for 12 h [6]. The authors pointed out the correlation between the diameter of particles and the drug release profile. The prolonged release time was observed for particles whose diameter was higher. The system of PLGA–auranofin particles-in-hydrogel was obtained first by the formation of particles using a nanoprecipitation method and then by dropping = their aqueous suspension into the thermogelling mixture.

Besides solid particles, nanocapsules, hollow spherical particles displaying high loading capacity, were also suspended in a hydrogel frame. Clotrimazole loaded in nanocapsules composed of Eudragit^®^ RS100 in Pemulen^®^ TR1-based hydrogel resulted not only in the enhancement of clotrimazole’s solubility but also in the extension of clotrimazole’s release from the nanocapsules-in-hydrogel, i.e., 20.14 ± 2.33 µg/cm^2^, in comparison to the rate of drug release from the hydrogel with entrapped free drug (70.63 ± 6.20 µg/cm^2^). As a result, the nanocapsule-enriched hydrogel ensured continuous, prolonged drug release.

The valuable pharmacological activity of substances of natural origin makes them alternatives to synthetic drugs; thus, there is growing interest in incorporating them into a hydrogel matrix. For example, hydrophobic curcumin, a polyphenolic compound [48], mainly extracted from the rhizome of turmeric, displays anti-inflammation activity [49]. A hydrophilic dispersion of curcumin in poly (vinyl pyrrolidone) was embedded in an in-situ-formed hydrogel based on Poloxamer, a thermogelling polymer [50]. The hydrogel displayed a strong intravaginal antibacterial effect on both *Escherichia coli* and *Staphylococcus aureus*. Although the curcumin-loaded hydrogel displayed weak in vitro antibacterial activity, satisfactory in vivo action was observed, which was explained by local immune regulation and the improvement of *Lactobacillus* growth. Moreover, the hydrogel displayed wound-healing properties attributed to curcumin.

Chitosan-based hydrogel with an embedded nanoemulsion of *Pelargonium graveolens* essential oil displayed higher antifungal activity in comparison to both an essential oil dilution and nanoemulsion, as the attained minimum inhibitory concentration value was 64 times lower [33]. The better antifungal activity of oil nanodroplets in the hydrogel resulted from the mucoadhesive properties of chitosan, which guaranteed mucosal penetration, enhancing the interactions between microorganisms and the oil nanoemulsion. A comparable enhancement in antifungal activity was also detected for a chitosan-based hydrogel enriched with *Mitracarpus frigidus* extract [32]. A 10% formulation exhibited greater and faster antifungal activity, as 50% of the fungus load was reduced at the third day after treatment started, whereas, with a higher concentration of neat *Mitracarpus frigidus* extract, only 70% of the fungal burden was diminished. The efficient adhesion of the chitosan-based hydrogel to the vaginal mucosa improved the extract delivery and thus enhanced the antifungal properties. A *Mitracarpus frigidus* extract-based hydrogel formulation did not show in vivo acute or sub-chronic toxicity and thus is not harmful for the vulvovaginal system.

Resveratrol, a polyphenol substance of natural origin that displays anti-*Chlamydia trachomatis* activity, has highly limited bioavailability due to poor water solubility. The incorporation of resveratrol into liposomes, however, resulted not only in improved solubility but also stability. Resveratrol-based liposomes were obtained by dissolving resveratrol in ethanol and mixing it with Lipoids S100 (phosphatidylcholine) dissolved in methanol. After the organic solvent had been evaporated, water was added and the whole content was shaken, yielding liposomes. The prepared drug carrier was embedded in a chitosan hydrogel. Resveratrol release from the liposome–hydrogel formulation was prolonged. In addition, 30% of resveratrol was released after 8 h, whereas, from resveratrol-based liposomes, 50% of the drug was released within this time.

The characteristics of hydrogel systems equipped with synthetic or natural pharmacologically active compounds for gynaecological therapies are listed in Table 1.

## 4. Drug-Free Hydrogels Displaying Antimicroorganism Action

Proper selection of the elements contained in the hydrogel can guarantee the achievement of a therapeutic platform with antimicrobial activity, without introducing typical anti-infection drugs. Chitosan is the gold standard in this field. It is well-known that it displays antimicrobial activity, including fungal pathogenic species for humans, such as *Candida* [54,55] and Gram-negative bacteria [56]. The antifungal activity possibly results from damage to the membranes of pathogens and the leakage of intracellular constituents, as an effect of interactions between chitosan protonated amine groups and negatively charged cell surface proteins [57].

The influence of chitosan additives on the hydrogel in the form of a monomolecular state and in the form of nanoparticles using a low-molecular-weight polymer was investigated. Perinelli et al. demonstrated that the hydrogel system composed of hydroxypropyl cellulose with free chitosan additive was active against both albicans and non-albicans strains, whereas the system enriched with assembled chitosan nanoparticles displayed anti-*Candida* activity against only non-albicans strains [58]. In terms of intrinsic activity, no evident differences between hydrogels prepared with free chitosan or in the form of nanoparticles, however, were observed. Joraholmen et al [56]. demonstrated activity against both *Staphylococcus epidermidis* and *Staphylococcus aureus* for a 0.1% chitosan-based hydrogel. It is noteworthy that liposomes coated with chitosan at the same concentration were merely active against *Staphylococcus epidermidis.* The concentration of chitosan introduced onto liposomes necessary to achieve activity against *Staphylococcus aureus* was 0.3%. The higher required concentration of chitosan was explained by the spherical shape of the liposomes, leading to the reduced number of electrostatic interactions between chitosan and the negatively charged bacterial cell membrane.

The incorporation of chitosan into the material with a synthetic polymer constituent resulted in the achievement of antiprotozoan activity. Semi-synthetic core–shell polymer nanoparticles composed of a hydrophobic core of poly (isobutylcyanoacrylate) (PIBCA) and a chitosan shell with thiol moieties embedded in a thermoresponsive Pluronics^®^ F127-based hydrogel exhibited strong anti-*Trichomonas vaginalis* activity [59]. Since neither chitosan in the solution nor pure PIBCA nanoparticles displayed any *Trichomonas* activity, the construct of chitosan-coated PIBCA nanoparticles was responsible for these antiprotozoan properties. It is likely that the anti-*Trichomonas* activity results from electrostatic interactions between the positively charged shells of the particles with the negatively charged protozoan membrane thanks to the presence of sialic acid [60]. The Pluronics solution with dispersed nanoparticles was able to form a mucoadhesive hydrogel film on the vaginal mucosa at the vaginal temperature. The close hydrogel adhesion ensured the maintenance of high local nanoparticle concentrations at the vaginal epithelium surface.

Metal nanoparticles, thanks to their intrinsic antimicrobial activity, were also applied to form hydrogel-based therapeutic platforms dedicated to gynaecological treatment. Silver nanoparticles were embedded in a hydrogel by the initial preparation of a particle suspension in an aqueous mixture of Poloxamer 407, Poloxamer 188, and Carbopol, which, after administration into the vagina in the form of a foam aerosol, was gelled upon the increase in temperature [24]. This hybrid material demonstrated antibacterial activity against P. aeruginas, S. aureus, and E. coli and moderate activity against C. albicans. In addition, it displayed significantly lower irritation of the vaginal tissue in comparison to the silver nanoparticle suspension.

An interesting direction in the development of intravaginal hydrogel-based therapeutic platforms lies in the combination of the pharmacological properties of both the polymer building components and drug activity, resulting in a synergistic effect on the pharmacological action. For example, a dual mechanism of antibacterial activity was demonstrated for a hydrogel composed of thiopyridyl-ended poly (amidoamine) dendrimer and thiol-terminated 8-arm poly (ethylene glycol) loaded with amoxicillin [38]. Besides the action of the introduced drug, additional antimicrobial activity was generated by the amine-terminated dendrimer, as an effect of the antibacterial activity by alteration of the bacterial cell wall [38,61]. The total release decreased in inverse proportion to the weight fraction of the components used for the hydrogel formation. This behaviour was strictly related to the reduction of hydrogel swelling resulting from the higher cross-linking density of the polymer network. The higher the polymer fraction used for the hydrogel formation, the lower the pore size observed. A synergy in anti-inflammatory and antibacterial activity was observed for epicatechin (natural polyphenol)-based liposomes-in-hydrogel built from chitosan [46]. Chitosan boosted the anti-*Candida* activity along with *Thymbra capitata* essential oil incorporated into a low-molecular-weight chitosan hydrogel [51]. Enhanced antifungal activity was also achieved in a chitosan-based hydrogel system enriched with *Pelargonium graveolens* essential oil [33].

The characteristics of drug-free hydrogels exhibiting antimicrobial activity are listed in Table 2.

## 5. Bioadhesive Nature of Hydrogels for Gynaecological Therapies

Interfacial forces existing between two materials, i.e., both biological or one biological and the second synthetic, are called bioadhesion. The extent of bioadhesion determines the extension of the period of time for which they are held together [62]. Amongst interactions governing bioadhesion, mainly ionic, hydrogen, hydrophobic, or van der Waals bonds can be distinguished [63]. In the case of drug delivery systems for topical gynaecological therapy, bioadhesion refers to the adhesion between the vaginal tissue and the drug platform. Sufficient drug platform retention on the afflicted area is crucial to attain a proper therapeutic effect by the efficient transfer of active substances to the tissue. A longer residence time for a therapeutic carrier can improve the mucosal penetration, contributing to an increase in drug bioavailability and thus leading to a reduction in the application frequency and dose. For example, enhanced antifungal activity was observed for a chitosan hydrogel thickened nanoemulsion containing *Pelargonium graveolens* essential oil. Chitosan improved drug delivery to *Candida* cells, allowing electrostatic interactions between the nanoemulsion and microorganisms, which led to enhanced antifungal properties. The mucoadhesive properties of polymer-building hydrogels are not only important in prolonging the residence time of the formulation in the vagina, but also in the enhancement of antimicrobial activity as a result of the improved adhesion of drug molecules to the afflicted area [32].

The bioadhesive properties of drug formulations are also of great importance in view of the vagina’s self-cleaning mechanism, which decisively shortens the residence time of the pharmacological platform. Longer retention of the hydrogel can be achieved by proper choice of mucoadhesive building components [64]. The mechanisms of bioadhesion between the mucosa and the hydrogel platform strictly depend on the polymer/s building the hydrogel. Both natural and synthetic polymers can be bioadhesive. The presence of chitosan in hydrogel systems increases the mucoadhesion behaviour. The chitosan mucoadhesion results from interactions with negatively charged groups, such as carboxylate (COO^−^) and sulfonate (SO_3_^−^) moieties present in mucine [65], hydrogen bonding, and hydrophobic effects [66]. In addition, chitosan has the capacity to interact with epithelial tight junctions, increasing drug penetration. An analogous mechanism to chitosan’s mucoadhesion regards ionic interactions of Eudragit^®^ RS100, a synthetic copolymer. Generally, the electrostatic interactions are formed between negatively charged mucin thanks to sialic acid moieties and cationic groups of Eudragit^®^ RS100 [35]. Cationic ions coming from the chlorotrimethylammonioethyl groups of the polymer are in contact with negatively charged mucin; thus, the transfer of electrons, creating an electrical double layer at the polymer/mucin interface, is promoted. Namely, the mucoadhesion mechanism consists in the attraction forces of the electrical double layer.

Gellan gum, a natural polysaccharide, is also considered a mucoadhesive polymer due to the formation of hydrogen bonds between the carboxyl groups of glucuronic acid, the hydroxyl groups of the gellan gum, and the appropriate H-group donor/acceptor groups of the mucin [63,67]. Another natural polymer, i.e., gelatin, a protein derived from animal sources such as bovine, porcine, and fish, by collagen hydrolysis, displays mucoadhesion thanks to the presence of hydrophilic groups such as carboxyl, hydroxyl, and amine, which can participate in hydrogen bonding [68]. Amongst synthetic polymers whose adhesion is governed by the ability to form hydrogen bonds with polysaccharides present in the mucosal lining, Carbopol^®^ 934, an acrylic acid-based polymer, must be mentioned [69]. Analogous behaviour is exhibited by Pemulen^®^TR1, a high-molecular-weight poly (acrylic acid) [53].

Poly (ethylene oxide)-b-poly (propylene oxide)-b-poly (ethylene oxide) (PEO-PPO-PEO) block copolymers (Pluronics, Poloxamers) bind to the oligosaccharide chains of the mucosal membrane with hydrophilic oxide groups [70,71]. Generally, a higher fraction of hydrophilic oxide groups in the copolymer results in a higher bioadhesive force. For example, the addition of PF68, a homologue PF127, resulted in an increase in bioadhesion, since 80% of PF68 was composed of hydrophilic oxide groups.

Thiolated polymers display adhesive properties via the interactions of thiol moieties with cysteine-rich domains of mucus via the formation of disulphide linkages [72]. This mechanism was exploited in the case of a hydrogel constructed from thiolated carboxymethyl hyaluronic acid [41] or a star-shaped poly (ethylene oxide) bearing thiol end group [38].

In some cases, a combined effect of mucoadhesive properties can be attained in a material constructed from two mucoadhesive components, which has been demonstrated for gellan gum- or Carbopol-based hydrogels equipped with Eudragit^®^ RS100 nanocapsules [35].

## 6. Conclusions and Perspectives

The development of intravaginal drug carriers is of great important to avoid the systemic exposure effects associated with oral administration, as most orally administered antibacterial medicines result in numerous side effects. Local, noninvasive drug administration is especially recommended for pregnant women and those who suffer from gastrointestinal tract problems. Moreover, intravaginal drug delivery ensure the avoidance of the first pass of the metabolism and thus protection of the drug against gastrointestinal enzymatic degradation. Further progress in the area of hydrogel-based gynaecological platforms should result in more efficient therapies that ensure complete recovery, without the recurrence of infection, after the administration of a single dose.

The gynaecological disorders are, however, often complex. For example, infections caused by pathogens of various origin may be accompanied by hormonal dysregulation. Therefore, there is a need to develop complex hydrogel platforms, carrying two or more drugs, with a sustained (e.g., for a week) and often different release profile. To achieve this goal, the relationship between the structural properties of the hydrogel system, such as cross-linking density, diffusivity of the network, mucoadhesion, in vivo degradation, interactions between drug and polymer network, etc., in view of their pharmacological efficiency in the vaginal environment must be determined in detail. An in-depth understanding of these factors is of crucial importance in designing an optimal intravaginal drug delivery system for women at different stages of life (adolescence, pregnancy, menopause, or post-menopausal period, etc.), taking into account the pathologies of the vagina, such as vulvovaginitis or cervical cancer. The vaginal environment changes, e.g., during pregnancy, during the menopausal period, or during infection; thus, the influence of such factors as pH on the structural properties of hydrogel systems has to be taken into account during the design of hydrogel platforms dedicated to gynaecological therapies.

Therefore, to achieve the ultimate goal of improving women’s health and quality of life, a holistic approach to the design of hydrogel-based therapeutic platforms for gynaecological treatment is required that involves developments in polymer chemistry, physics, and biomedical engineering.

## Figures and Tables

**Table 1:** The characteristics of antimicrobial polymer-based hydrogel systems for gynaecological treatment.

Composition/Type of Hydrogel	Drug	Infection Type	Characteristics	Ref.
Carbopol^®^ 934-based hydrogel with embedded sesame oil organogel	Metronidazole	Bacterial vaginosis	- Formation of oil-in-water emulsion gel (10–35 µm droplet oil phase size), - Shear-thinning flow behaviour, - Diffusion mediated drug release	[36]
Thiolated carboxymethyl hyaluronic acid cross-linked with poly(ethylene glycol)-bisbromoacetate	Metronidazole/ metronidazole benzoate	Bacterial vaginosis	- Flexible mucoadhesive films that swells upon rehydration in vaginal conditions, - Hydrogel retention for 6 days, - Wound healing and anti-inflammatory properties	[41]
Pluronic^®^ F127/chitosan-based hydrogel	Metronidazole	*Trichomonas vaginalis*	- Thermoresponsive hydrogel - a controlled release of drug (68% of released drug after 24 h)	[29]
Gellan gum and Carbopol hydrogels containing nanocapsules composed of Eudragit^®^ RS 100 shell and rosehip oil-based core	Indole-3-carbinol (I3C)	*Trichomonas vaginalis*	- Increased anti-Trichomonas vaginalis activity of I3C upon nanoencapsulation in Eudragit^®^ RS100 - Mucoadhesive properties of liposomes and gel forming polymer - Bettter mucoadhesion of Gellan gum-based hydrogels than Carbopol-based systems, - Shear-thinning properties	[35]
*Pelargonium graveolens* oil-based nanoemulsion thickened with low molecular chitosan	*Pelargonium graveolens* essential oil	Candidiasis	- Mucoadhesive properties, - 64 times improved antifungal activity of hydrogel formulation against *C. albicans* and *C. glabrata*, 32 times for *C. krusei* and 16 times for *C. parapsilosis* in comparison to essential oil dilution and to nanoemulsion as a result of chitosan presence in the hydrogel	[33]
Resveratrol-based liposomes-in-chitosan hydrogel	Resveratrol	Bacterial vaginosis	- Anti-*Chlamydia trachomatis* activity; - Anti-inflammatory action; - Sustained drug release for 8 h; - Safe formulation for pregnant women (no systemic effect)	[45]
Chitosan	*Mitracarpus frigidus* methanolic extract, MFM	Vaginal candidiasis	- Higher and faster antifungal activity of hydrogel drug-loaded platform in comparison to neat MFM extract - Shear-thinning properties	[32]
Chitosan	Natural polyphenol resveratrol,	Bacterial vaginosis (*Chlamydia trachomatis*)	- Enhanced anti-chlamydial effect upon resveratrol incorporation in liposomes embedded in the hydrogel, - Strong in vitro, anti-inflammatory activity - Retention of resveratrol on the tissue (the lack of its systemic effect) - Sustained drug release	[45,46]
*Thymbra capitata* essential oil incorporated into low molecular weight chitosan hydrogel	*Thymbra capitata* essential oil	Vaginal candidiasis	- Chitosan enhances both anti-*Candida* activity and mucoadhesion	[51]
*Thymus vulgaris* essential oil embedded in polycarbophil-based hydrogel	*Thymus vulgaris* essential oil	Vaginal candidiasis	- Antifungal activity against *Candida*	[52]
Curcumin solid dispersion stabilized with poly(vinyl pyrrolidone) embedded in Poloxamer hydrogel	Curcumin	Bacterial vaginosis	- In situ gelling - Anti-*Escherichia coli*, and *Staphylococcus aureus* activity; - 71.6% curcumin release in 24 h	[50]
Gelatin/PEG hydrogel	Metronidazole	Bacterial vaginosis	- Mucoadhesive properties thanks to gelatin, - Antimicrobial activity against *E.coli* and *E. subtilis*, - Swelling dependent on the composition, i.e., a decrease in water uptake of the hydrogels with the increase in PEG fraction, - The release profile can be modulated with the mutual weight fraction of gelatin and PEG in the hydrogel. The higher weight fraction of PEG in the hydrogel composition resulted in the decreased release, which was associated with reduced macromolecular relaxation of the polymer chains in the hydrogel, and thus lower water migration in the hydrogel frame	
Pemulen (high molecular weight polyacrylic acid derivative)/Pellulan hydrogel enriched with drug-loaded Eudragit^®^ RS100-based nanocapsules	Clotrimazole	Candidiasis	- Mucoadhesive properties thanks to Pullulan and Eudragit® RS100, - Drug retention on the mucosa surface	[53]
Drug saturated hydroxypropyl β-cyclodextrin nanosponges embedded in Pluronic^®^ F-127 and Pluronic^®^ F-68-based hydrogel	Clotrimazole	Candidiasis	- In situ gelling in 60 s., temperature-dependent increase in viscosity of the gel (from 1 × 10^3^ cP at 25 °C to 2 × 10^5^ cP at 37 °C) - In vivo antifungal activity, - In vitro bioadhesion	[28]
drug-loaded poly(lactic-co-glycolic acid) nanoparticles, PLGA embedded in the hydrogel composed of β-glycerophosphate and chitosan	Auranofin	*Trichomonas vaginalis*	- Sol-gel transition within a minute upon temperature shifting from 20 to 37 °C - Mucoadhesive properties - Sustained drug release for 12 h, - High topical drug efficacy thanks to the synergistic effect of sustained drug release from PLGA and prolonged nanoparticles retention in the vaginal tissue, - Higher in vivo efficacy of auranofin-loaded hydrogel in comparison to orally-administered high doses, - No apparent liver and topical toxicity	[6]
Poloxamer^®^ 407/chitosan lactate hydrogel and chitosan lactate/glycerophosphate-based hydrogel	Amoxicilin	Bacterial vaginosis	- Comparable drug release profiles (45% of released drug after 3 h) for both hydrogel formulations, - Chitosan lactate/glycerophosphate-based hydrogel displayed better antimicrobial and wound healing properties, - Better mucoadhesion of chitosan lactate/glycerophosphates system than Poloxamer^®^ 407/chitosan lactate hydrogel - An addition of chitosan lactate to Poloxamer increased the gelation point from 30 to 37 °C - Dilution with simulated vaginal fluid resulted in the increase in the gelation time from 100 to 500 s	[26]
Poloxamer^®^ 407	Amphotericin B	Vaginal candidiasis	- Sol-gel transitions at 32 °C - Gel can be administered as an aerosol - Sustained drug release for 24 h, - Non-irritant, - No change in skin alteration was observed, - Regeneration of ulcerated skin	[23]
Thiopyridyl-ended poly(amidoamine) [G4-(NH_2_)_64_ dendrimer and thiol-terminated 8-arm poly(ethylene glycol)	Amoxicilin	Bacterial vaginosis	- Gelling in 10–30 s after mixing both polymer components, - Degrades in the presence glutathione-enriched cervical secretions, - Dual antibacterial action, i.e., thanks to drug and amine-terminated dendrimer properties, - Sustained amoxicilin delivery for more than 240 h - Safe for the treatment of infections of pregnant women	[38]
Star-shaped poly(furfuryl glycidyl ether)-block-poly(glyceryl glycerol ether) and poly(2-acrylamidephenylboronic acid-*ran*-acrylamide)	Nifuratel	Bacterial vaginosis, candidiasis and *Trichomonas vaginalis*	- Boronic ester cross-links formation at vaginal pH - Injectable, - Self-healing properties, - 80% release of entrapped drug in 10 h	[42]

Eudragit^®^ RS100—a copolymer of ethyl acrylate, methyl methacrylate, and chlorotrimethylammonioethyl methacrylate. Poloxamer—poly (ethylene oxide-b-propylene oxide-b-ethylene oxide). Polycarbophil—poly (acrylic acid) cross-linked with divinyl glycol and with calcium as a counter-ion.

**Table 2:** Examples of polymer-based hydrogel systems displaying intrinsic antimicrobial properties.

Composition/Type of Hydrogel	Infection Type	Characteristics	Ref.
Medium molecular weight chitosan-based hydrogel (M_n_ = 270,000, 86% deacetylated)	Candidiasis	- Thermosensitive mechanism of gelling; - Antifungal properties: 50% of *C. albicans* cells were killed upon 5 mg/mL chitosan concentration for 1 h. Complete inhibition of *Candida tropicalis*, *Candida krusei*, *Candida guilliermondii* and *Candida parapsilosis*	[57]
Medium molecular weight chitosan	Bacterial vaginosis	- Acid-induced gelation, - The hydrogel thanks to chitosan intrinsic displayed activity against *Staphylococcus epidermidis* and *Staphylococcus aureus* at 0.1 wt% polymer concentration	[56]
Nanoparticles composed of poly(isobutylcyanoacrylate), PIBCA core and a chitosan and thiolated chitosan-based shell	*Trichomonas vaginalis*	- Thermoresponsive mechanism of gelling; - Mucoadhesive properties thanks to the presence of chitosan and thiolated chitosan in the nanoparticles’ shell, - Anti-*Trichomonas vaginalis* activity was thanks to poly(isobutylcyanoacrylate) nanoparticles coated with chitosan	[59]
Free chitosan or chitosan nanoparticles embedded in hydroxypropyl methylcellulose	Candidiasis	- Anti-*Candida* activity of hydrogel embedded with free chitosan against both albicans and non-albicans strains; - Hydrogel with chitosan nanoparticles displayed only anti-*Candida* activity on non-albicans species	[58]
Silver nanoparticles in the suspension with Poloxamer^®^ 407, Poloxamer^®^ 188 and Carbopol^®^ 974P	Bacterial vaginosis	- An expansible thermal gelling aerosol, - Antibacterial activity against *P. aeruginas*, *S. aureus* and *E. coli* and moderate activity against *C. albicans*, - Significantly lower irritation to vaginal tissue in comparison to silver nanoparticles suspension	[24]
Thiopyridyl-ended poly(amidoamine)dendrimer and thiol-terminated 8-arm poly(ethylene glycol)	Bacterial vaginosis	- Synergistic effect of antibacterial activity assured by the amine terminated dendrimers and incorporated amoxicillin	[38]
Low-molecular weight chitosan	Bacterial vaginosis	- Acid-induced gelation, - Anti-inflammatory properties, - Anti-bacterial activity	[51]
Low-molecular weight chitosan	Candidiasis	- Acid-induced gelation, - Synergistic activity against *Candida*	[33]

## Data Availability

Not applicable.
